# Experimental status epilepticus, COX‐2 and BDNF: Connecting the dots

**DOI:** 10.1002/epi4.12501

**Published:** 2021-06-16

**Authors:** Nicola Marchi

**Affiliations:** ^1^ Cerebrovascular and Glia Research Institut de Génomique Fonctionnelle (University of Montpellier, CNRS UMR5203, INSERM U1191) Montpellier France

Brain and peripheral inflammation and immune pathways play distinct roles in a variety of epilepsies. Disease modification(s) and reduction in seizure burden by targeting specific pro‐inflammatory mediators have represented a demanding and controversial quest.^1^, ^2^, ^3^ Within this realm, the inducible cyclooxygenase‐2 (COX‐2) and brain‐derived neurotrophic factor (BDNF), and their signaling cascades, were proposed as relevant candidate entry points in central nervous system (CNS) disorders.^2^, ^4^, ^5^, ^6^ COX‐2 is upstream to, and controls, a broad inflammatory pathway leading to the production of prostaglandin E2 (PGE2). As COX‐2 brain expression increases during seizures, the effectiveness of COX‐2 inhibitors has been tested with varying outcomes.^2^

Here, Yu and Jiang have outlined previously missing links and the temporal dynamics of COX‐2 and BDNF expression in the hippocampus in response to status epilepticus (SE).^7^ To avoid possible model‐specific biases, they elicited experimental SE by injecting either KA or pilocarpine in rodents. With a panel of molecular biology tools, they showed that, in the hippocampus, the induction of COX‐2 temporally preceded that of BDNF and that blocking COX‐2 activity with a specific inhibitor (SC‐58125) prevented the BDNF surge post‐SE. Next, in proceeding to validate their hypothesis: i) They found that PGE2, a key COX‐2 product, was sufficient to promote BDNF secretion from hippocampal cells; and ii) they targeted the PGE2 receptor EP2 by using a selective, brain‐permeable antagonist (TG6‐10‐1) that subsequently decreased downstream BDNF and TrkB (BDNF receptor) signaling in the hippocampus post‐SE. From these data, they conclude that, after experimental SE, COX‐2 controls BDNF/TrkB signaling via PGE_2_/EP2. Because the EP2 antagonist TG6‐10‐1 reduced BDNF/TrkB signaling, the authors propose this pharmacological strategy to limit the inflammatory pathophysiology typical of post‐SE in these models.

These results support the general concept that an early and targeted pharmacological intervention after an acute brain insult (here SE^8^) may curb the pathological sequel over time, here epileptogenesis leading to spontaneous seizures. Because of the post‐SE rapidly evolving pathophysiological traits, identifying or targeting a specific culprit is challenging, particularly when it comes to inflammation and the varying, even contrasting, roles that specific cell types or soluble factors may have as a function of time, brain regions, and tissue lesion. Furthermore, when considering specific factors and ​their receptors, the extent of supra‐threshold stimulation(s) should be considered. Thus, the activation of BDNF is required for a variety of physiological processes including learning and memory,^9^ while an excessive BDNF/TrkB interplay, or activation, plays a pathological role in certain brain diseases including epilepsies.^4^ These delicate and varying equilibriums need to be controlled when planning the ensuing large‐scale (pre)clinical trials and in case of clinical applications, along with a methodical examination of systemic and brain unwanted side effects associated with pro‐ or anti‐inflammatory modifications or adaptations.^1^, ^10^, ^11^, ^12^

In conclusion, this work connects COX‐2 activation to BDNF/TrkB and PGE2/EP2 in the hippocampus after experimental SE. The authors propose the use of a brain‐permeable antagonist as a strategy to suppress the abnormal BDNF/TrkB activity with a hypothesized preclinical application to post‐insult settings.

Read the winning article—https://doi.org/10.1002/epi4.12409

## CONFLICTS OF INTEREST

I have no conflicts to disclose. I confirm that I have read the Journal's position on issues involved in ethical publication and affirm that this report is consistent with those guidelines.
